# Non-pharmacological interventions for Lewy body dementia: a systematic review

**DOI:** 10.1017/S0033291717003257

**Published:** 2017-11-16

**Authors:** Michael H. Connors, Lena Quinto, Ian McKeith, Henry Brodaty, Louise Allan, Claire Bamford, Alan Thomas, John-Paul Taylor, John T. O'Brien

**Affiliations:** 1 Sydney Medical School, University of Sydney, Sydney, NSW, Australia; 2 Dementia Centre for Research Collaboration, UNSW Sydney, Sydney, NSW, Australia; 3 Centre for Healthy Brain Ageing, UNSW Sydney, Sydney, NSW, Australia; 4 Institute of Neuroscience, Newcastle University, Newcastle, United Kingdom; 5 Institute of Health and Society, Newcastle University, Newcastle, United Kingdom; 6 Department of Psychiatry, University of Cambridge, Cambridge, United Kingdom

**Keywords:** Caregiver support, dementia with Lewy bodies, Lewy body dementia, neuropsychiatric symptoms, non-pharmacological, Parkinson's disease dementia

## Abstract

Lewy body dementia (consisting of dementia with Lewy bodies and Parkinson's disease dementia) is a common neurodegenerative disease characterised by visual hallucinations, fluctuating attention, motor disturbances, falls, and sensitivity to antipsychotics. This combination of features presents challenges for pharmacological management. Given this, we sought to review evidence for non-pharmacological interventions with patients with Lewy body dementia and their carers. Bibliographic databases were searched using a wide range of search terms and no restrictions were placed on study design, language, or clinical setting. Two reviewers independently assessed papers for inclusion, rated study quality, and extracted data. The search identified 21 studies including two randomised controlled trials with available subgroup data, seven case series, and 12 case studies. Most studies reported beneficial effects of the interventions used, though the only sizeable study was on dysphagia, showing a benefit of honey-thickened liquids. Given the heterogeneity of interventions and poor quality of the studies overall, no quantitative synthesis was possible. Overall, identified studies suggested possible benefits of non-pharmacological interventions in Lewy body dementia, but the small sample sizes and low quality of studies mean no definite recommendations can be offered. Our findings underscore the clear and urgent need for future research on this topic.

## Introduction

Lewy body dementia is a common neurodegenerative disease in older people (Walker *et al.* 2015). It is responsible for 5–25% of diagnosed cases of dementia (Vann Jones & O'Brien, 2014), giving it a likely prevalence of around 1% in people over 65 years old (Ballard *et al.* 2013). The disease is characterised by fluctuations in attention and alertness, recurrent visual hallucinations, and parkinsonian motor features (McKeith *et al.* 2005). It is broadly considered to consist of two related disorders – dementia with Lewy bodies and Parkinson's disease dementia – that are distinguished by the relative timing of when cognitive and motor symptoms appear. Dementia with Lewy bodies is diagnosed if dementia develops either prior to parkinsonian motor symptoms or within 1 year of their onset. By contrast, Parkinson's disease dementia is diagnosed if dementia develops after 1 year of parkinsonian motor symptoms. The two disorders share a common underlying pathophysiology, but likely vary in the sequence by which brain areas underpinning cognition and motor function are affected (Aarsland *et al.* 2009; McKeith, 2009).

Lewy body dementia poses a number of challenges for clinical management that differ from those of other dementias. People with Lewy body dementia typically experience visual hallucinations and may be more likely to have delusions than people with other types of dementia (Ballard *et al.* 1999; Aarsland *et al.* 2007; Brodaty *et al.* 2015). These types of symptoms can be very distressing to both patients and their carers, and are a risk factor for early institutionalisation (Black & Almeida, 2004; Brodaty *et al.* 2014). Pharmacological management, however, is limited because of the condition's adverse sensitivity to neuroleptics (antipsychotic drugs) (McKeith *et al.* 1992; Aarsland *et al.* 2005; Ballard *et al.* 2013). Antidepressants also appear to be poorly tolerated (Culo *et al.* 2010) with no clear evidence that they are effective for treating mood disturbances in this population (Ballard *et al.* 2013; Stinton *et al.* 2015), though further research is needed. At the same time, patients typically have parkinsonian motor disturbances and treatment responses are limited by the fact that some antiparkinson medications can exacerbate psychotic symptoms (McKeith *et al.* 2005). People with Lewy body dementia are also prone to falls, which further increases the risks of using medications that exacerbate orthostatic hypotension, impair cognition, or otherwise predispose to falls. Finally, people with Lewy body dementia may undergo faster cognitive decline (Rongve *et al.* 2016), require higher costs of care (Murman *et al.* 2003; Boström *et al.* 2007; Vossius *et al.* 2014), result in greater caregiver burden (Svendsboe *et al.* 2016), and progress more quickly to death (Oesterhus *et al.* 2014) than people with Alzheimer's disease and other dementias.

Despite these challenges and the relatively high prevalence of the disease, the evidence base for intervention strategies remains unclear. A previous systematic review and meta-analysis of pharmacological treatments for Lewy body dementia identified a lack of high-quality evidence for commonly used drugs in this population, though found evidence for efficacy of cholinesterase inhibitors (Stinton *et al.* 2015). Another systematic review evaluated the benefits of exercise in Lewy body dementia and likewise found little high-quality research (Inskip *et al.* 2016). There is, however, no synthesis of evidence for non-pharmacological options more generally for people with Lewy body dementia. Such options have been shown to be helpful in other types of dementia (Brodaty & Arasaratnam, 2012; Livingston *et al.* 2014; Orgeta *et al.* 2015), but are likely to be especially important in Lewy body dementia given the risks of medications in this population. In this paper, we addressed this issue and reviewed studies that assessed non-pharmacological interventions for patients with Lewy body dementia.

## Method

The protocol for this systematic review was registered at PROSPERO (registration number CRD42016050492).

### Eligibility criteria

The review focused on primary research that assessed a non-pharmacological intervention for either patients with Lewy body dementia or carers of such patients. The review considered studies evaluating non-pharmacological interventions in the broader categories of dementia or Parkinson's disease if the results of a Lewy body dementia subgroup were available. Studies that were confounded by a concurrent pharmaceutical intervention (i.e. medication initiated at the same time as a non-pharmacological intervention or given to one group in a study but not the other) were excluded. There were no other restrictions on study design and no requirements for a comparator group. There were also no restrictions on language, time period, or clinical context in which the study was conducted.

### Search strategy

The search identified studies through bibliographic databases, trial registers, and the grey literature. Bibliographic databases and trial registers included the following: Medline (1946–present); PreMedline, PubMed; EMBASE (1974–present), Scopus, Web of Science (1900–current); PsychInfo (1806–present); CINAHL (1981–present); Cochrane libraries: Cochrane database of systematic reviews (2005–October 2016), Cochrane central register of controlled trials (August 2016), Cochrane Methodology register (3rd–Quarter 2012); other EBM databases: ACP journal club (1991–September 2016), Database of Abstracts of Reviews of Effects (1st-quarter 2015), Health technology assessment (3rd-quarter 2016), and NHS economic evaluation (1st-quarter 2015); Ageline (1978–present); ALOIS; AMED (Allied and Complementary Medicine; 1985–present); PEDro (Physiotherapy Evidence Database; 1929–present); Social work Abstracts (1968–present); and the National Association of Social Workers (NASW) clinical register (14th edition). The grey literature was searched using such resources as SIGLE (System for Information on Grey Literature in Europe), NTIS (National Technical Information Service) database, and PsychEXTRA (1908–present).

The search strategy used only population and intervention terms to maximise the likelihood of identifying relevant studies (comparator and outcome terms were not used). The population was people with Lewy body dementia or their carers. This was identified using the search terms: [(Lewy OR Park*) and Dementia]. Interventions were any non-pharmacological treatment and identified using a wide range of terms: (activit*, acupuncture, alternative, animal, aromatherapy, art therapy, assisted, balance, behav*, bicycle, calisthenics, carer intervention, caregiver intervention, CBT, Chi gong, cognit*, cognitive behavioral therapy, cognitive behavioural therapy, counsel*, creative arts, dance, dancing, diet, direct current stimulation, drama, ECT, educat*, electroconvulsive therapy, enhanc*, environmental intervention, environmental modification, exercise, flexibility, humor therapy, humour therapy, hydrotherapy, intervention*, leisure, light therapy, management, martial arts, massage, meditation, Montessori, multisensory, music, non-pharm*, nonpharm*, nutrition, occupational therapy, pet therapy, physical activity, physical therapy, physiotherapy, pilates, psychoeducation, psychol*, psychosocial, psychotherapy, Qi gong, reality orientation, recreation*, reminiscence, resistance training, run*, sensory, simulated presence, stimulation, Snoezelen, support*, support group*, swim*, tai chi, therap*, therapeutic activity, TMS, training, training carers, training caregivers, transcranial magnetic stimulation, treatment*, validation, weight training, yoga). Searches were conducted on 30 October 2016.

In addition to bibliographic database searches, the reference lists of papers included in the review and previous systematic reviews on both Lewy body dementia and non-pharmacological interventions were checked for relevant papers. Advice was also sought from experts in the field.

### Study selection

Two reviewers (MHC and LQ) independently assessed search results for inclusion by title and abstract. All articles deemed relevant by either reviewer were obtained in full. Both reviewers then independently evaluated full-text articles for inclusion. Any disagreements were resolved through discussion or, if necessary, with a third reviewer (JTO).

### Data extraction

Two reviewers independently extracted relevant data from publications using a standardised form. This included participant details (e.g. demographics, number, recruitment, clinical context, dementia severity), intervention type, study design, measures, and results. Qualitative data were also collated.

The primary outcomes were measures of cognition, function, neuropsychiatric symptoms, and motor symptoms. The secondary outcomes were measures of any other clinically relevant outcomes, such as quality of life, carer burden, financial costs, other symptoms (sleep or autonomic disturbances), and objective endpoints (e.g. falls, hospitalisation, institutionalisation, mortality). Secondary outcomes also included the perceived acceptability of treatments, reported side effects, and dropout rates (a measure of treatment acceptability).

### Quality assessment

Two reviewers independently assessed study quality and risk of bias using standardised tools. These included the Effective Public Health Practice Project Quality Assessment Tool for Quantitative Studies (Effective Public Health Practice Project, 2007; Armijo-Olivo *et al.* 2012) and the NICE Methodology Checklist: Qualitative Studies (NICE, 2016). Any disagreements were resolved through discussion.

### Data synthesis

Although a quantitative synthesis of findings using meta-analytic techniques was originally intended, this was not possible due to the small sample sizes and poor quality of the included studies. As a result, only a descriptive synthesis was provided.

## Results

### Search results

**Fig. 1.**
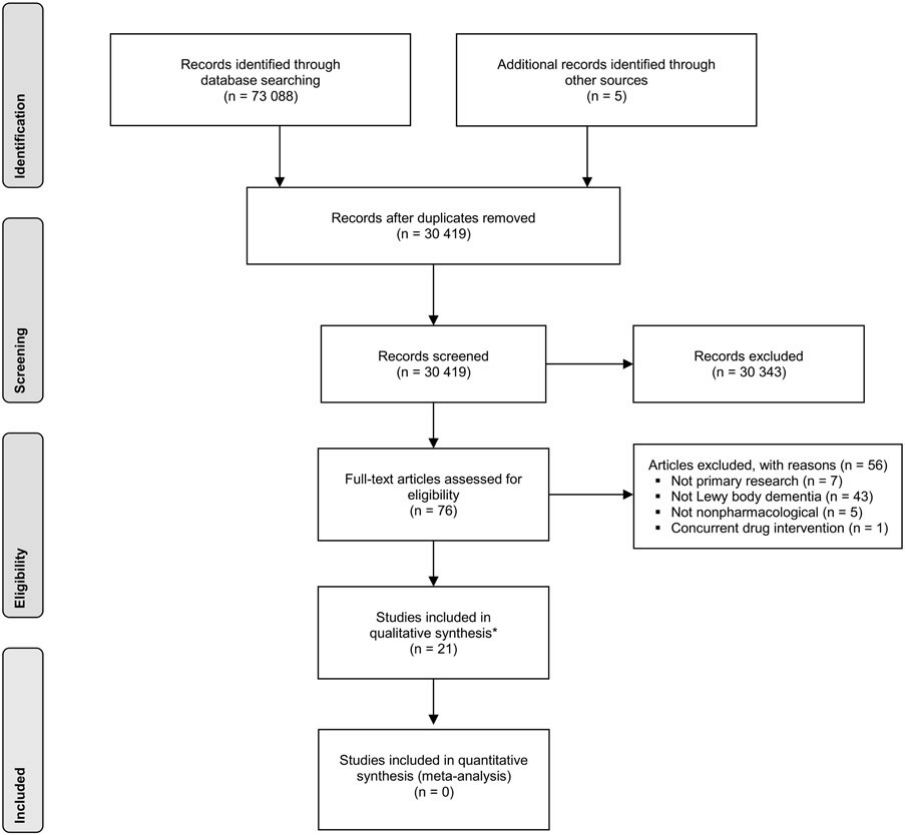
PRISMA flow chart for study selection. *Two of the studies were reported in the same article.

The search identified 73 093 publications, of which 30 419 were unique and 42 674 were duplicates. Of these, 76 were considered by one or both reviewers to be potentially relevant and obtained in full. In turn, 21 studies were found eligible for inclusion (a flow diagram in PRISMA format is shown in Fig. 1). The included studies consisted of 12 case studies (Kung & O'Connor, 2002; Loher *et al.* 2002; Graff *et al.* 2006; Cheston *et al.* 2007; Huh *et al.* 2008; Freund *et al.* 2009; Ciro *et al.* 2013; Gil-Ruiz *et al.* 2013; Tabak *et al.* 2013; Dawley, 2015; Hsu *et al.* 2015; Ricciardi *et al.* 2015), seven case series (Rasmussen *et al.* 2003; Rochester *et al.* 2009; Takahashi *et al.* 2009; Ota *et al.* 2012; Elder *et al.* 2016; Yamaguchi *et al.* 2016) – two of which were reported in the same paper (Takahashi *et al.* 2009) – and two randomised controlled trials (Logemann *et al.* 2008; Telenius *et al.* 2015). Both randomised trials focused on the broader conditions of dementia and/or Parkinson's disease but had separate results for a Lewy body dementia subgroup available. One case report was reported in two separate papers (Freund *et al.* 2009; Barnikol *et al.* 2010); our descriptive synthesis focused on the more detailed of these papers (Freund *et al.* 2009). A further case report on electroconvulsive therapy was excluded (Fujiwara *et al.* 2004) because of a concurrent pharmacological intervention. Twelve studies focused on dementia with Lewy bodies (Kung & O'Connor, 2002; Rasmussen *et al.* 2003; Cheston *et al.* 2007; Huh *et al.* 2008; Takahashi *et al.* 2009; Ota *et al.* 2012; Ciro *et al.* 2013; Gil-Ruiz *et al.* 2013; Hsu *et al.* 2015; Telenius *et al.* 2015; Yamaguchi *et al.* 2016), eight focused on Parkinson's disease dementia (Loher *et al.* 2002; Graff *et al.* 2006; Logemann *et al.* 2008; Freund *et al.* 2009; Rochester *et al.* 2009; Tabak *et al.* 2013; Dawley, 2015; Ricciardi *et al.* 2015), and one included patients from both groups (Elder *et al.* 2016). A summary of studies is shown in Table 1.

**Table 1.** Studies assessing a non-pharmacological intervention for Lewy body dementia

Study	Intervention	Design	Participants	Main outcomes	Findings
Logemann *et al.* (2008)	Interventions to prevent aspiration of fluids in patients with dysphagia (three conditions: honey-thickened fluids *v.* nectar-thickened fluids *v.* chin-down posture; all patients received all three interventions)	Randomised controlled trial	132 PDD with dysphagia (from a sample of 711 patients with dysphagia and either dementia or Parkinson disease)	Aspiration (as measured by videofluorographic swallow studies)	Honey-thickened fluids were superior to both nectar-thickened fluids and chin-down posture in reducing aspiration. Overall, 59% of patients aspirated when given honey-thickened fluids, 64% of patients aspirated when given nectar-thickened fluids, and 69% of patients aspirated when put in a chin-down posture. Pairwise comparisons were statistically significant (*p* < 0.01)
Telenius *et al.* (2015; data from Inskip *et al.* 2016)	Exercise (high-intensity functional exercises two sessions/week for 12 weeks *v.* control group)	Randomised controlled trial	Four DLB (from a sample of 170 patients with dementia)	Gait speed, balance, other physical measures, function	Patients in the exercise group showed signs of improved gait speed. The small number of participants with Lewy body dementia in each condition and missing data prevented between-group statistical comparisons
Tabak *et al.* (2013)	Exercise (stationary cycling; three sessions/week for 8 weeks)	Case study	One PDD (from a sample of two patients with Parkinson disease)	Cognition, function, quality of life, gait speed	The patient showed improvements in cognition, quality of life, and gait speed
Rochester *et al.* (2009)	Interventions to improve gait (auditory cueing of gait with a metronome and verbal instructions in a single session)	Case series	Five PDD (from a sample of nine patients with Parkinson disease)	Gait speed, stride amplitude	Cues that focused attention on temporal aspects (stepping to the metronome's beat) and spatial aspects (taking large steps) appeared to improve gait speed and stride amplitude
Dawley (2015)	Physical therapy (Lee Silverman voice treatment-big intervention; eight sessions over 3 months)	Case study	One PDD	Gait speed, balance, function	The patient showed some improvements in gait speed, balance, and functional ability despite poor compliance with the intervention
Huh *et al.* (2008)	Multi-component intervention including carer education (32 × 1 h sessions) and tailored environmental modification	Case study	One DLB	Agitation, function, carer burden	The patient displayed less agitation. The carer reported less distress. Functional measures, although collected, were not reported
Ota *et al.* (2012)	Psychological intervention for visual hallucinations, including psychoeducation and environmental modification (duration and details not specified)	Case series	Two DLB	Anxiety, frequency and content of hallucinations	Patients reported reduced anxiety around hallucinations and that hallucinations were less frequent (abstract only; details not provided)
Gil-Ruiz *et al.* (2013)	Environmental modification for mirrored self-misidentification delusion (reducing the mirror size and personalising it with artwork)	Case study	One DLB with delusion	Signs of the delusion, patient's distress at mirror	The patient did not display the delusion in front of the modified mirror (when the modifications were inadvertently removed, the delusion returned; when the modifications were replaced, the delusion again subsided)
Hsu *et al.* (2015)	Music therapy (one interactive session tailored to the patient)	Case study	One DLB	Agitation	Patient showed reduced agitation and anxiety after the session according to the experimenter
Cheston *et al.* (2007)	Simulated presence (two sessions)	Case study	One DLB (from a sample of six patients with dementia)	Distress	Patient showed less distressed behaviour (e.g. less frequent asking to go home) and more prosocial behaviour (e.g. talking calmly with others) after the sessions
Ciro *et al.* (2013)	Occupational therapy (‘skill building through task-oriented motor practice’; STOMP; five sessions/week for 2 weeks)	Case study	One DLB	Function (3 tasks: ability to stand from a recliner, put on eyeglasses, and brush teeth)	The patient improved in her abilities to stand from a recliner and put on her eyeglasses. The patient, however, showed no improvement in her ability to brush her teeth
Graff *et al.* (2006)	Occupational therapy (system-based intervention focused on improving the patient's functional abilities and his carer's behaviours)	Case study	One PDD	Function, quality of life, qualitative reports of both patient and carer	The patient showed some improvement in function and reported greater autonomy and quality of life. His carer reported improved communication with the patient and better understanding of the condition
Kung & O'Connor (2002)	Electroconvulsive therapy (unilateral; seven sessions)	Case study	One DLB	Depression, other neuropsychiatric symptoms	Patient demonstrated less depression and fewer neuropsychiatric symptoms for 2 weeks after treatment. These benefits, however, were not sustained thereafter
Rasmussen *et al.* (2003)	Electroconvulsive therapy (mostly bilateral, though details varied with patient)	Case series	Seven DLB	Depression	Participants reported less depression after treatment; two participants also reported less frequent hallucinations. Group data were not reported. Longer term outcomes were unclear
Yamaguchi *et al.* (2016)	Electroconvulsive therapy (details not provided)	Case series	Six DLB	Depression, psychotic symptoms, motor function (details not provided)	Patients showed less depressive and psychotic symptoms after treatment, as well possibly reduced motor symptoms (Abstract only; group data and details not provided)
Takahashi *et al.* (2009)	Electroconvulsive therapy (bifrontotemporal; six sessions)	Case series	Eight DLB with depression	Depression	Patients reported lower depression scores on the Hamilton Depression Rating Scale after treatment (before treatment, mean = 38.0, s.d. = 5.8; after treatment, mean = 15.0, s.d. = 9.6; *p* < 0.01)
Takahashi *et al.* (2009)	Transcranial magnetic stimulation (dorsolateral prefrontal cortex bilaterally; 10 sessions)	Case series	Six DLB with depression	Depression	Patients reported lower depression scores on the Hamilton Depression Rating Scale after treatment (before treatment, mean = 24.0, s.d. = 8.0; after treatment, mean = 11.0, s.d. = 5.9; *p* < 0.01)
Elder *et al.* (2016)	Transcranial direct current stimulation (single 20 min session)	Case series	Five DLB, Eight PDD	Neuropsychological battery focused on attention and visuoperceptual abilities	Patients showed improvements in some measures of attention after treatment (patients reported more correct answers on a choice reaction time task, *d*_*z*_ = 0.83, and showed faster reaction times on a digit vigilance task, *d*_*z*_ = 0.80). There were no changes in other measures of attention or visuoperceptual performance
Freund *et al.* (2009; also described in Barnikol *et al.* 2010)	Deep brain stimulation (two electrodes in the nucleus basalis of Meynert; two electrodes in the subthalamic nucleus)	Case study	One PDD	Cognition, motor symptoms	Patient showed improved cognition and motor symptoms depending on the location stimulated: stimulating the nucleus basalis of Meynert was associated with better cognition and less apraxia, whereas stimulating the subthalamic nucleus was associated with less parkinsonian motor symptoms
Loher *et al.* (2002)	Deep brain stimulation (single electrode in the left internal segment of globus pallidus)	Case study	One PDD	Motor symptoms, cognition, and function	The patient's right-sided motor symptoms improved considerably. The patient's cognitive and functional abilities, however, continued to decline
Ricciardi *et al.* (2015)	Deep brain stimulation (unilateral stimulation of the pedunculopontine nucleus)	Case study	One PDD	Cognition	The patient's cognition continued to decline gradually. After 4 years, stimulation was turned off and the patient's cognition deteriorated significantly. Stimulation was then resumed and the patient's cognition returned to what it was immediately prior to switching stimulation off

DLB, dementia with Lewy bodies; PDD, Parkinson's disease dementia.

### Quality assessment

Of the 21 studies, 19 were rated as poor quality due to their small sample size, uncertainties about recruitment, and the lack of a control group. One randomised controlled trial, which focused on exercise in nursing homes for patients with dementia (Telenius *et al.* 2015), was evaluated as poor quality with respect to Lewy body dementia specifically (it only included four participants with the condition and these participants did not complete all tasks) (Inskip *et al.* 2016). The other randomised controlled trial (Logemann *et al.* 2008), which investigated dietary fluid and postural interventions for dysphagia, was evaluated as moderate quality. This study, however, was limited by its lack of blinding and the fact that it relied on measures of immediate efficacy in a research setting, without any longer term follow-up or measures of real-world effectiveness. It was also limited by the absence of a control group that did not receive a treatment, making it difficult to establish the absolute effectiveness of the interventions.

### Participants

The total number of participants across the included studies was 195. This comprised 44 patients with dementia with Lewy bodies and 151 patients with Parkinson's disease dementia (of whom, 132 were from one study; Logemann *et al.* 2008). Demographic information was not consistently reported. It was not provided for the subgroup of 132 patients with Parkinson's disease dementia in the largest study (Logemann *et al.* 2008) and for two case series (Ota *et al.* 2012; Yamaguchi *et al.* 2016) – altogether 140 participants. Of the data available (*n* = 55), the mean age of participants was 70.6 years (s.d. = 8.2) and included 27 males and 28 females. Measures of patients’ cognition and dementia severity were lacking from the majority of reports. Of the 21 studies, nine recruited participants from nursing homes or hospitals, five recruited participants from the community, and seven did not report participants’ residential status. One of the studies that recruited participants from nursing homes examined the effect of an intervention on both a patient with dementia with Lewy bodies and her carers (Huh *et al.* 2008).

### Interventions

Interventions included carer education (Huh *et al.* 2008), psychological interventions (for visual hallucinations) (Ota *et al.* 2012), physical exercise (Tabak *et al.* 2013; Telenius *et al.* 2015), gait cueing (Rochester *et al.* 2009), environmental modification (Gil-Ruiz *et al.* 2013) (for mirrored self-misidentification, a delusion that usually occurs in the context of dementia; Connors & Coltheart, 2011; Connors *et al.* 2015), music (Hsu *et al.* 2015), simulated presence (Cheston *et al.* 2007), occupational therapy (Graff *et al.* 2006; Ciro *et al.* 2013), physical therapy (Dawley, 2015), dietary fluid and postural interventions to prevent aspiration (Logemann *et al.* 2008), electroconvulsive therapy (Kung & O'Connor, 2002; Rasmussen *et al.* 2003; Takahashi *et al.* 2009; Yamaguchi *et al.* 2016), transcranial magnetic stimulation (Takahashi *et al.* 2009), transcranial direct current stimulation (Elder *et al.* 2016), and deep brain stimulation (Loher *et al.* 2002; Freund *et al.* 2009; Ricciardi *et al.* 2015). The most studied interventions were electroconvulsive therapy (four studies) and deep brain stimulation (three studies).

### Effectiveness of interventions

Given the heterogeneity of interventions and the poor quality of the research evidence, no quantitative synthesis was possible. A descriptive summary of individual studies is provided in Table 1.

All studies reported some effectiveness of their respective intervention. The strongest evidence came from the randomised control trial that assessed interventions to prevent fluid aspiration in participants with dysphagia (Logemann *et al.* 2008). This trial compared honey-thickened fluids, nectar-thickened fluids, and a chin-down posture in 132 patients with Parkinson's disease dementia (see Table 1). It found that the use of honey-thickened fluids was superior to the other two methods in preventing fluid aspiration as measured by videofluorographic swallow studies.

Four small uncontrolled studies (Kung & O'Connor, 2002; Rasmussen *et al.* 2003; Takahashi *et al.* 2009; Yamaguchi *et al.* 2016) – involving 22 participants in total – found that electroconvulsive therapy had some effectiveness in treating depression in dementia with Lewy bodies. In one of these studies, two patients exhibited confusion immediately after electroconvulsive therapy (Rasmussen *et al.* 2003); in another study, an unspecified number displayed signs of autonomic dysfunction, though without any lasting effects (Takahashi *et al.* 2009). No other adverse events were reported. Another three case studies of deep brain stimulation (Loher *et al.* 2002; Freund *et al.* 2009; Ricciardi *et al.* 2015) identified benefits in either cognition or motor symptoms depending on the location of stimulation in patients with Parkinson's disease dementia. All three studies used different brain locations for stimulation. None of these studies reported adverse effects.

Other individual studies variously reported benefits of carer education for reducing agitation (Huh *et al.* 2008); psychological interventions for reducing distress around visual hallucinations (Ota *et al.* 2012); physical exercise to improve gait and function (Tabak *et al.* 2013; Telenius *et al.* 2015); gait cueing to improve gait speed (Rochester *et al.* 2009); environmental modification to reduce the distress associated with mirrored self-misidentification delusion (Gil-Ruiz *et al.* 2013); music therapy to reduce agitation (Hsu *et al.* 2015); simulated presence to reduce distress (Cheston *et al.* 2007); occupational therapy interventions to improve functional ability (Graff *et al.* 2006; Ciro *et al.* 2013); physical therapy to improve gait (Dawley, 2015); dietary fluid and postural interventions to prevent aspiration (Logemann *et al.* 2008); transcranial magnetic stimulation to reduce depression (Takahashi *et al.* 2009); and transcranial direct current stimulation to improve attention (Elder *et al.* 2016) (see Table 1). For all these studies, however, limitations in their design, including small sample sizes, lack of blinding, and lack of adequate controls, mean that it is not possible to rule out confounding, selection bias, experimenter expectancy, publication bias, and other causes for the reported effects.

## Discussion

The systematic review identified very little research on non-pharmacological interventions in Lewy body dementia. No randomised control trials focusing specifically on Lewy body dementia were identified and the majority of research consisted of case studies and case series. Of the studies identified, the best quality evidence came from a randomised control trial that focused on preventing fluid aspiration in Parkinson's disease patients with dysphagia (Logemann *et al.* 2008). As already noted, however, even this study was limited by the fact that it only examined the immediate effects of interventions in an experimental context and did not assess longer term effectiveness in more naturalistic settings. Overall, given the heterogeneity of interventions, small sample sizes, and poor quality of research, no treatment recommendations can be offered.

The lack of research in this area is in contrast to the large amount of research on non-pharmacological interventions in other types of dementia (Brodaty & Arasaratnam, 2012; Livingston *et al.* 2014; Orgeta *et al.* 2015; Livingston & Cooper, 2017; Seeher & Brodaty, 2017). It is consistent, though, with what has been found in other systematic reviews on Lewy body dementia (Hindle *et al.* 2013; Stinton *et al.* 2015; Inskip *et al.* 2016). Despite the lack of research, however, non-pharmacological interventions are likely to be important in the management of Lewy body dementia. As already noted, pharmacological treatment of psychotic symptoms and movement disturbances in this condition is limited by the difficulties patients have tolerating first-line medications.

At the same time, there is evidence for the effectiveness of non-pharmacological interventions for similar symptoms in other conditions. In the case of psychosis, for example, multi-factorial interventions, including physical activity, occupational therapy, and music therapy, have some evidence of effectiveness in other types of dementia (Brodaty & Arasaratnam, 2012; Chen *et al.* 2014). In the case of motor disturbances, interventions such as exercise and gait training have been found to be effective in Parkinson's disease (Bloem *et al.* 2015). There is also strong evidence that non-pharmacological interventions are effective at ameliorating symptoms that are common across different dementias (Brodaty & Arasaratnam, 2012; Livingston *et al.* 2014; Orgeta *et al.* 2015; Seeher & Brodaty, 2017). Caregiver education, training, and support, for example, have been shown to reduce both carers’ distress and patients’ neuropsychiatric symptoms (Brodaty & Arasaratnam, 2012; Seeher & Brodaty, 2017). Likewise, psychological interventions, such as cognitive behavioural therapy, have been shown to reduce patients’ depression (Orgeta *et al.* 2015). Finally, organised activities, music therapy, and sensory stimulation have been shown to reduce patients’ agitation (Livingston *et al.* 2014). All of these interventions could be investigated more systematically in Lewy body dementia.

Altogether, the review highlights the clear and urgent need for research in this area. Possible barriers to this include challenges establishing the diagnosis and distinguishing it from other dementias (McKeith *et al.* 2005; Vann Jones & O'Brien, 2014); the limited research resources devoted to Lewy body dementia as opposed to other neurodegenerative conditions; and the general lack of funding for non-pharmacological interventions. Each of these, however, is not insurmountable. The use of multi-centre registries or networks of centres with expertise in Lewy body dementia, for example, could help to address the problem of recruitment. Given the prevalence of the disease, its disproportionately high social and economic burden (Murman *et al.* 2003; Boström *et al.* 2007; Vossius *et al.* 2014; Svendsboe *et al.* 2016), and the limited suitability of medications, future research into non-pharmacological interventions is important to improving management at both individual and population levels.
